# “I get hungry all the time”: experiences of poverty and pregnancy in an urban healthcare setting in South Africa

**DOI:** 10.1186/s12992-015-0122-z

**Published:** 2015-08-25

**Authors:** Fiona Scorgie, Duane Blaauw, Tessa Dooms, Ashraf Coovadia, Vivian Black, Matthew Chersich

**Affiliations:** Centre for Health Policy/MRC Health Policy Research Group, School of Public Health, Faculty of Health Sciences, University of the Witwatersrand, Johannesburg, South Africa; Wits Reproductive Health & HIV Institute (WRHI), Faculty of Health Sciences, University of the Witwatersrand, Johannesburg, South Africa; Department of Sociology, University of Johannesburg, Johannesburg, South Africa; Department of Paediatrics and Child Health, Faculty of Health Sciences, University of the Witwatersand, Johannesburg, South Africa

**Keywords:** South Africa, Pregnancy, Poverty, Antenatal care, Nutrition, Social determinants of health

## Abstract

**Background:**

For pregnancy to result in a healthy mother and infant, women require adequate nutrition and to be able to access antenatal care, both of which require finances. While most women working in the formal sector in South Africa obtain some form of maternity leave, unemployed women receive no such support. Additional interventions in the form of expanded social assistance to vulnerable pregnant women are needed. To help inform such an approach, we undertook a series of qualitative interviews with low-income pregnant women in Johannesburg.

**Methods:**

Qualitative, in-depth interviews were held with 22 pregnant women at a public sector antenatal clinic in Johannesburg in 2011 to gather data on their greatest needs and priorities during pregnancy, their access to financial resources to meet these needs, and the overall experience of poverty while pregnant.

**Results:**

A total of 22 women were interviewed, 5 of whom were primagravid. One woman was in the first trimester of pregnancy, while nine were almost full-term. All but one of the pregnancies were unplanned. Most participants (15/22) were unemployed, two were employed and on paid maternity leave, and the remaining five doing casual, part-time work. In most cases, pregnancy reduced participants’ earning potential and heightened reliance on their partners. Women not living with the father of their children generally received erratic financial support from them. The highest monthly expenses mentioned were food, accommodation and transport costs, and shortfalls in all three were reportedly common. Some participants described insufficient food in the household, and expressed concern about whether they were meeting the additional dietary requirements of pregnancy. Preparing for the arrival of a new baby was also a considerable source of anxiety, and was prioritized even above meeting women’s own basic needs.

**Conclusions:**

Though pregnancy is a normal life occurrence, it has the potential to further marginalise women and children living in already vulnerable households. Extending the Child Support Grant to include the period of pregnancy would not only serve to acknowledge and address the particular challenges faced by poor women, but also go some way to securing the health of newborn children and future generations.

## Background

Income poverty and inequality remain fundamental problems in South Africa, and many households have insufficient resources to meet their needs. In 2009, Statistics South Africa estimated that 52.3 % of the population were living below the upper poverty line of ZAR577 per person per month ($72) [1]. 1 Although overall levels of food insecurity have declined in the past decade, under-nutrition remains a serious problem [2], with about half of South African households experiencing hunger and a further third at risk of it should their income decline [3]. The state’s welfare programme, primarily providing unconditional cash transfers to the caregivers of poor children and to old-age pensioners, plays a vital role in improving household food security, but in practice these interventions often fail to reach the poorest households [4].

As in many other low- and middle-income countries (LMICs), poverty in South Africa follows the fault-lines of gender inequality. Census figures from 2000 show that income and expenditure in male-headed households was just over double that of households headed by women [5]. Despite some positive shifts in gender relations, the transition to democracy has not brought the change in this area that was so widely anticipated. Writing in 2005, Goldblatt noted that women were still less likely to be employed than men and have lower paying jobs than men. She concluded that “[t]he sexual divisions within the workplace, home and the society as a whole remain largely untouched by the many changes that have occurred in the last decade” [6].

Data gathered by Statistics South Africa for the national General Household Survey (GHS) build a telling picture of how pregnant women in particular are disadvantaged by this gendered system [7]. In the 2010 survey, roughly a quarter of pregnant women reported earning an income or running their own business, compared to about half of all adults in South Africa. Even compared to other women of reproductive age, pregnant women were 45.6 % less likely to have an income. A quarter of pregnant women (26.6 %) lived in households earning under ZAR800 (US$100) per month. Most pregnant women (66.9 %) nationally resided in households receiving a social grant, with a quarter living in households that had experienced food insufficiency in the past year. Levels of food insecurity, measured by having missed meals or reduced meal sizes, were also considerably higher in the households of pregnant women than other households. Importantly, this link between pregnancy and poverty has been found in other settings, even in high-income countries. A study of low-income pregnant women in 19 states in the USA identified surprisingly high levels of poverty and food insecurity around the time of pregnancy, and found that childbearing women have considerably lower incomes than do women of childbearing age overall. It also found that serious hardships – such as divorce, separation, homelessness and job loss – were very common for poor women during this life period [8].

When women are poor, their pregnancies are likely to be negatively impacted in several ways: they are at high risk of malnutrition, and by extension, their infants are vulnerable to nutritional and developmental deficiencies [9]. The growth demands of pregnancy require a substantial increase in maternal macronutrient consumption, much of which is essential for normal foetal development. Poor nutritional status during pregnancy, as indicated by a low body mass index of women, short stature, anaemia, or other micronutrient deficiencies, increases the likelihood of obstructed labour, caesarean delivery and postpartum haemorrhage [9]. Poor maternal nutrition also raises the risk of intrauterine growth restriction, having a baby with a low birth weight and other adverse pregnancy outcomes, as well as impaired neonatal growth and cognitive development later in a child’s life [10, 11]. Furthermore, it is increasingly being accepted that *in-utero* malnutrition is associated with long-term consequences for the individual, including conditions such as diabetes, obesity, hypertension, cardiovascular disease and abnormal cholesterol profiles (the ‘thrifty phenotype’ hypothesis) [12].

Financial and other barriers to accessing antenatal and obstetric services have been documented in South Africa [13], often reflecting a deep disempowerment created by poverty, whereby women are unable or reluctant to claim their rights to health care. Late attendance of antenatal care services has been documented both in an inner-city clinic in Johannesburg [14] and in a rural area in KwaZulu-Natal [15], raising concerns about initiation of antiretroviral therapy (ART) early enough to reduce maternal mortality and paediatric HIV infection. Sub-optimal use of maternal health services has been identified as a critical cause of maternal deaths in South Africa [16]. This may go some way towards explaining why the country has a substantially higher maternal mortality rate than its middle-income status would otherwise predict, and is unlikely to meet the Millennium Development Goal for reducing maternal mortality [17].

As the information above suggests, our knowledge of how poverty impacts on pregnancy is considerably biomedical and quantitative in orientation, with a strong focus on the cumulative, population-level effects of malnutrition and the sub-optimal use of maternal health services. Little research has been done on the experiential dimensions of pregnancy for women living in poverty; many of these studies have a strong focus on psychological consequences of poverty. For example, a phenomenological study of vulnerable women in Chile during pregnancy and in the postpartum focused predominantly on the sense of hopelessness and despair they experienced during this time [18]. Other studies have explored associations between poverty and depression in low-income settings both during and after pregnancy [19, 20]. We know from a substantial body of research that nutritional needs increase during pregnancy, but when household resources are severely limited, how do women manage these additional food demands? Beyond nutrition, what other needs are women faced with as they prepare for the arrival of their newborn? And what does it mean for poor women when these needs cannot be met?

Such evidence gaps have important policy implications. To date, the only form of state intervention specifically targeting pregnant women in South Africa has been the removal of user-fees for health antenatal and childbirth services. Arguably, there is a need for considering additional structural interventions in the form of expanded social assistance to pregnant women. To help inform such an approach, we undertook a series of qualitative interviews with women attending antenatal services in a public-sector hospital in Johannesburg. In this small descriptive study, we seek to understand the key financial needs experienced by women during pregnancy, how women prioritise these increased needs and the extent to which they deepen vulnerability when unmet.

## Methods

In-depth interviews with 22 pregnant women were held in the antenatal clinic of Rahima Moosa Mother & Child Hospital in December 2011. The hospital is a public-sector facility providing secondary-level paediatric and obstetric services, and is situated in an economically deprived suburb close to the inner-city of Johannesburg. These data were collected as part of a broader study commissioned by the national Department of Social Development to assess levels of vulnerability among pregnant women in poor households and the need for direct state intervention to ameliorate this vulnerability. Approval for study activities was obtained from the Human Research Ethics Committee (Medical) of the University of the Witwatersrand (no. M110946).

The hospital’s patient population encompasses women of different races and language groups, although black African and ‘coloured’ women predominate. As a public-sector hospital, most patients attending the clinic come from households categorised as lower-income; in South Africa, only those with financial means have the health insurance required to access private healthcare.

### Recruitment of participants

We interviewed participants until the total planned sample size of 22 was achieved. The patient files belonging to patients attending the antenatal clinic each day were screened by clinic nurses for study eligibility. The eligibility criteria included: South African citizenship (as state grants only presently cover citizens) and being 18 years and older. We did not exclude potential participants on the basis of their income level. Ten eligible patient files were then chosen at random. From this pile, every second file was selected until three had been identified for the day’s interviews. The patients corresponding to these files were then approached individually in the queue by the nurses, who briefly introduced the researchers and explained the nature of the study. If the patient indicated a willingness to participate, they were accompanied to a private room and the study explained in more detail by the researchers. The explanation included information on the potential risks and benefits of participation, and that, given the sensitive nature of the enquiry, they could decline to answer any question during the interview. Patients agreeing to participate had an opportunity to ask questions and gave informed consent. Those declining to participate returned to the queue and additional files were selected from the original ten patient files until we reached the full quota of three interviews per day. Over the course of the study, only two patients declined participation.

Midway through the selection process, the research team reviewed the socio-demographic characteristics of the participants already interviewed, and found that women older than 30 years of age were being over-sampled. For the remainder of the study, we therefore modified the selection procedure described above and chose to purposively recruit the remaining patients (i.e. selecting younger women to approach), in order to obtain a more representative sample of women of different ages.

### Interview procedures and data analysis

All interviews were held in a private room within the antenatal clinic. Two members of the research team were present during interviews, with one leading the interview while the other asked additional questions or probes, and extracted data from patient files. In seven instances, a translator with experience as a research fieldworker in multilingual contexts joined the team for interviews in seSotho or isiZulu. Four interviews with Afrikaans-speaking participants were conducted by one member of the research team who was fluent in Afrikaans.

All but one interview were recorded using a digital recorder and later transcribed. One participant withheld permission for the recorder to be used; for this interview, handwritten notes were taken. Interviews lasted 30 to 60 minutes and were semi-structured, following a set of broad questions on themes such as: greatest needs (financial and otherwise) while pregnant; barriers to meeting these needs; interactions with health services; eating patterns before and during pregnancy; and socio-economic background and degree of empowerment within the household. At the close of each interview, a brief socio-demographic questionnaire was completed, capturing quantitative information on educational background, income, employment and household composition.

**Fig. 1:**
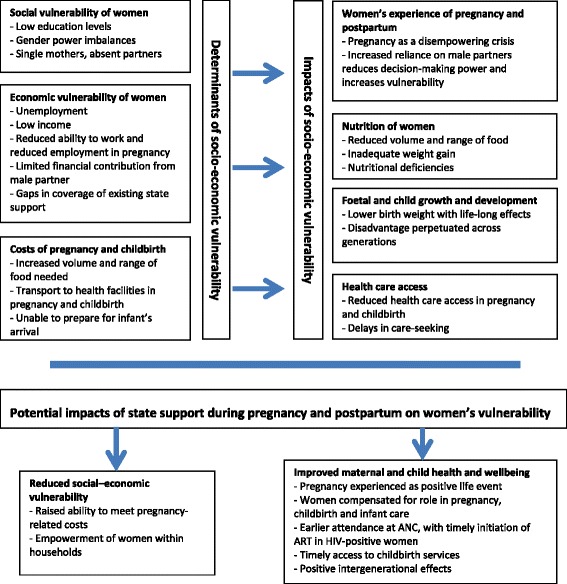
Conceptual framework showing determinants and impacts of socio-economic vulnerability among pregnant women

Socioeconomic vulnerability was assessed by examining factors such as employment and income generation, receipt of state support, as well as level of education, gender of the household head, and financial and other contributions received from one’s partner. In this paper, poverty is defined as living below the upper poverty line of ZAR577 per person per month ($72), as mentioned above. Participants whose income placed them above this poverty line and whose narratives clearly did not reflect a position of socioeconomic vulnerability were nevertheless included in the analysis. Contrasting the experiences of these women with poorer ones provided useful insights, and helped to show how, at least in some way, how state support for poorer women might alter their health and wellbeing during pregnancy. After identifying the main themes emerging from interview transcripts, manual coding was undertaken and consensus on a final set of themes was reached through discussion among the research team. The findings are summed in the text, together with illustrative quotes. A conceptual framework was developed to depict how the interacting determinants of socio-economic vulnerability impact on women’s experiences of pregnancy and its outcomes (Fig. 1). This framework also shows how state support for pregnant women might mitigate the effects of women’s vulnerability in pregnancy.

## Results

### Profile of study participants

#### Demographics and pregnancy history

A total of 22 women were interviewed (see Table 1). In terms of age, this sample was largely reflective of the national distribution as recorded in the GHS (see Additional file 1: Table S1 [7]), with 16 of the 22 participants aged 20–29 years and three women younger than 20. More than half (12) had only completed primary school, with many having started but not finished secondary level. All participants were either black African or ‘coloured’ women.

**Table 1:** Selected socio-demographic and economic characteristics of pregnant women interviewed at Rahima Moosa Mother & Child Hospital

Variable	Total % (*n* = 22)
Age groups, in years	
18–24	32 (7)
25–29	55 (12)
30+	14 (3)
Highest education level	
Primary school	55 (12)
Secondary school	41 (9)
Tertiary	5 (1)
Had worked in past 7 days	
No	68 (15)
Marital status	
Married	30 (6)
Unmarried, living with partner	30 (6)
In relationship, not living with partner	35 (7)
Divorced	5 (1)
Number of previous children	
0	23 (5)
1–2	68 (15)
3-5	9 (2)
Number of people in household	
1–2	19 (4)
3–5	57 (12)
6+	24 (5)
Household income (ZAR/month)	
501–2500	38 (8)
2501–4500	14 (3)
4501–6000	14 (3)
8000-max	33 (7)
Individual income (ZAR/month)	
0	64 (14)
1–500	9 (2)
501–1500	14 (3)
2501-4500	14 (3)

Only 5 of the 22 participants were primagravid. One woman was in the first trimester of pregnancy, while nine were almost full-term. Given that Rahima Moosa Hospital is a referral centre for high-risk pregnancies, it was unsurprising that many of the women had experienced some medical complication during pregnancy. Although only 5 of the 22 participants were anaemic, eight were HIV positive (36 %), and several more reported a history of ‘high-risk’ pregnancies or other health conditions, such as diabetes, hypertension, or kidney problems. Importantly, all but one of the participants’ pregnancies were unplanned, and most of these were reportedly a consequence of contraceptive failures. Related to this, about a quarter of women had only found out that they were pregnant when already beyond four months into pregnancy. Some expressed shock, sadness and even anger at discovery of their pregnancy, partly because of the burden of unanticipated expenses in pregnancy and for the newborn.

### Socio-economic circumstances

Most participants were unemployed at the time of the interview (15 out of 22), two were formally employed and on paid maternity leave, and the remaining five participants were doing casual, part-time work such as braiding hair, selling goods in a market and packing boxes. This work was reportedly difficult to access or continue while pregnant, as employers were reluctant to hire pregnant women. Since the majority of participants had been unemployed for some time or had worked only in the informal sector, they were not eligible for benefits from the state’s unemployment insurance fund (UIF^2^),which is available exclusively to formal-sector employees.

Participants vividly described the sense of helplessness experienced when unemployed and when household resources were meagre and inadequate. A 24-year old woman pregnant with her third child explained:

*“The fact that I’m not working and also the fact that I don’t have money to buy the things I need to stay healthy is a hardship. Because sometimes when the grant money for my one child gets finished, things become very hard.”*

A second participant living in an informal settlement with her husband expressed repeated concern about how they would find the money to support another child.

*“[F]or now the most important thing for me might be the fact that I may not have enough money to support my child…I won’t have the power to buy the milk. I don’t have enough money for that” [25 year-old woman with 1 dependant, unemployed]*

Another said:

*“Like my boyfriend is there, but [he] isn’t working. I’m also not working, so there’s no-one that will be able to help…at the end when I give birth, to get clothing for the child” [29 year-old woman with 2 dependants, unemployed]*

In most cases, pregnancy reduced participants’ earning potential and heightened reliance on their partners. The only two women in the sample who reported being unemployed by choice at the time of becoming pregnant were in committed relationships with men who earned enough to meet the household’s needs. This contrasted strongly with the experience of the majority of women interviewed, for whom having a working partner was no guarantee of financial security.

*“…now I’m not working you see, and the money that my husband is getting is too little, the money for one person to pay rent like accommodation, buying food, buying baby’s food, transport to go to work, it’s difficult” [27 year-old married woman, unemployed].*

Participants who were not living with the father of their children generally received erratic financial support from them. This unpredictability created significant anxiety for these women, who then had to turn to others for support. One woman described receiving very little from the father of the child she was expecting, despite the fact that he had formal employment and a regular income:

*“…but he is not supportive that much…sometimes he gives me one hundred rand a month or sometimes he just buys me a couple of fruits.” [24 year-old woman, not living with partner, unemployed]*

Another recounted how she had resorted to legal means to try to secure reliable support from her older child’s father, in the form of regular maintenance payments rather than *ad hoc* contributions:

*“…because today he is working and another day he was not working, I had to be running to small courts in and out. So now since he is working we found out two months ago, he is not giving me cash like in a bank, like he is supposed to do. He is just buying his daughter outfits and giving us what he has got.” [23 year-old woman with 1 dependant, unemployed]*

When it came to decisions about household spending, those who were unemployed, and especially younger women, typically did not have the power to decide how money was spent in the home. Women who lived with their parent(s) even reported commonly handing over a part of or their entire wage to them for household expenses. This usually meant that decisions about money to be kept aside specifically for pregnancy-related expenses were made by the parents of the pregnant woman without her input at all. Two women described increased tension and conflict with sexual partners over the additional expenses triggered by the pregnancy. In the section below, we take a closer look at what these costs typically entailed.

### The cost of pregnancy

When asked to detail their typical monthly household expenses, women listed a wide range of items, including primarily: food, accommodation, transport, school expenses, toiletries, and cell-phone costs. Three items were mentioned consistently as the highest monthly expense across all households, namely, food (64 % of participants), accommodation (23 %) and transport costs (14 %), in other words, basic living expenses. Shortfalls in these areas, however, were common. In the sections that follow, we examine how these basic needs are magnified and rendered more complex when a woman in a poor household becomes pregnant.

### Nutrition: managing the disjuncture between need and affordability

Participants were asked what their average daily food intake was, and details captured on what had been consumed the day prior to the interview. The diverse responses indicated that intake of food during pregnancy is shaped not only by individual food preferences, the limitations imposed by pregnancy-related nausea, or medical conditions such as diabetes, but in large part by what is available given household financial constraints.

Twenty of the 22 participants reported increased appetite during pregnancy.

*“I get hungry all the time, so I need to get food all the time. It’s not the way it used to be before because I never used to get hungry like this and now when I get hungry, I cannot wait to eat later in the day as I used to wait before pregnancy. I must have something to eat right away.” [24 year-old woman with 2 dependants, unemployed]*

*“I used to eat once a day, now I have to eat three times a day or even more.” [19 year- old woman, part-time domestic worker]*

Two women reported appetite declines related to nausea and the onset of hypertension during pregnancy – but even in these cases, they emphasised the need to eat enough to ensure the health of the baby.

*“…I have to keep this child, so you have to eat.” [29 year-old woman with 1 dependant, unemployed]*

This increased nutritional intake was a challenge particularly where nausea and vomiting persisted beyond the first trimester. Participants who struggled with this mentioned the need for additional nutritional supplements – such as multi-vitamins or fortified drinks – which were either obtained from the clinic or purchased out-of-pocket.

In addition to the need for increased volumes of food, women spoke about the need to eat a wide range of foods or to satisfy particular cravings. Participants seemed well informed about the types of food and diversity considered essential for ensuring healthy and successful pregnancies. Eating the right kinds of food – fruit and vegetables, fish, chicken, and meat such as liver were listed – was frequently mentioned as desirable, often without prompting.

*“Fruits and veggies and also everything that has grains in it for the vitamins” [19 year- old woman, first pregnancy, part-time domestic worker]*

Several participants expressed concern about whether they were meeting the additional dietary requirements of pregnancy, since there was simply insufficient food available in the household.

*“…now I’m not working you see, and the money that my husband is getting is too little, the money for one person to pay rent like accommodation, buying food, buying baby’s food, transport to go to work, its difficult” [27 year woman with 1 dependant, unemployed]*

*“I try to eat healthy, but many a times you find that I need something, but find it very hard to get it. But each time I get food, I make sure to eat healthy so as to keep the baby healthy too.” [24 year old woman with 2 dependants, unemployed]*

In general, they described spending substantially more money on food than before pregnancy.

*“…before my pregnancy I used to eat anything that was available, but now I have to eat healthily, so now I have to spend more money on healthy foods than I was doing before.” [24 year-old woman with 2 dependants, unemployed]*

*“I spend more money because the things that I’m eating now are different from the things I used to eat back then… There are some things that I did not like back then that I love now, and because of that I now have to buy it.” [28 year-old woman with one dependant, unemployed]*

Virtually without exception, the daily intake of poorer participants included a very limited variety of food – consisting of little more than ‘pap’ (traditional porridge made from ground maize), gravy, one vegetable, bread and a piece of fruit, for example. One participant, who was unemployed, described her total intake of food on the day preceding the interview as:

*“… in the morning I ate an apple, and in the afternoon I ate bread and eggs, and then I also ate fruit later on when I was going to bed.” [24 year-old woman with 2 dependants, unemployed]*

She went on to explain that it was not necessarily possible even to eat this much every day:

*“it depends on the availability of food, I eat only in times I know that there is food, but if there is no food I just eat a fruit until I eat later on in the day.”*

And on days when there is literally no food in the house, she asks for *“help from the neighbours, so that I can get something to eat”.* A few relatively wealthier participants, on the other hand (those on paid maternity leave or living with partners who were employed), were able to combine a wider variety of vegetables and fruit, protein sources, a range of starches and dairy products, with ease.

### “I would buy baby stuff”: other economic needs unique to pregnancy

**Fig. 2:**
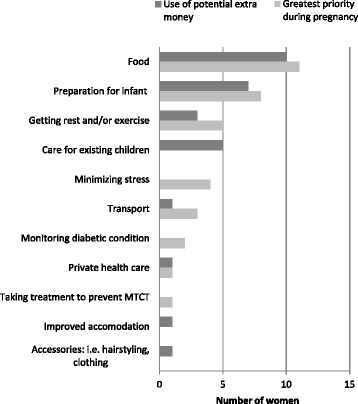
Participants’ greatest needs during pregnancy and how additional income would be spent (multiple response questions)

To assess more precisely the full range of economic needs of women during pregnancy and how these needs were prioritised, we asked participants if, hypothetically, they had around ZAR300-400^3^ extra each month (approx. US$37-50) for the duration of their pregnancy, how they would spend this cash (see Fig. 2, below). Their responses told us much about their most immediate unmet needs at this time. While food was, for most women, the first thing they would purchase with the extra money, buying items to prepare for the arrival of the baby was the next most popular response.

Preparing for the arrival of a new baby is a need unique to pregnancy, and for women without the means to support themselves and their families, it constituted a considerable source of anxiety. Most pressing were concerns about addressing the essential basic needs of the baby: food, clothing, diapers and suitable and secure accommodation.

*“I would buy clothes for the baby….I will use it for the baby’s needs” [29 year-old woman, first pregnancy, part-time domestic worker]*

*“The first thing I will spend it on, I don’t want to lie, is I will buy the preparation for the baby … So the first thing that I must do is to make sure that I have everything for the baby until the time I deliver…Things like clothes and if I’m not breastfeeding, buy supplementary milk. I mean formula for the baby because I don’t know if next year the government will still be issuing free milk I don’t know.” [25 year-old woman, HIV positive with 2 dependants, self-employed]*

*“Clothes for the kids, and I could use some of that three hundred on transport” [22 year-old woman, pregnant with first child, self-employed]*

*“Blankets, Pampers [nappies] and overalls for babies to stay warm.” [28 year-old woman with one dependant, unemployed]*

One woman mentioned also the need to specifically plan for the time of giving birth:

*“I would buy baby stuff, and stuff for myself like food so that I could be able to eat after I give birth. You find that other women don’t have anything to eat after giving birth, so I would buy food for me and my family to be able to eat after I give birth.” [19 year-old woman with 3 dependants, in part-time employment]*

In most cases, women insisted that items relating to “baby stuff” would be prioritized even above their own basic needs:

*“I also have many needs, such as food and clothes, but I would spend it on the things required by the baby.” [24 year-old woman with 2 dependants, unemployed]*

*“Sometimes I don’t have a Colgate [toothpaste]; I use Sunlight bath soap to wash my teeth or sometimes I don’t have a washing powder, I take that Sunlight soap to wash” [38 year-old woman with 3 dependents, unemployed]*

Many participants spoke about the pressures of having to provide income not only to cover current pregnancy-related expenses, but also to support other children in the household, who in some cases were still very young. Around a quarter of participants lived in households with more than five members. One unemployed woman spoke of the stress of having to buy formula milk for her 11-month old, while now also needing money to purchase *“stuff for the [new] baby”*. While a number of women were already accessing a Child Support Grant for their older children, and intended to apply for an additional grant for the new baby as well, these grants were often the only income source for the household. One woman, receiving a Child Support Grant and some income from the father of one of her children, explained how this combined income must stretch to cover not only her children’s schooling needs, but also buy food for other household members, including other children that are not her own. She gave an account of how this has impacted on her ability to feed herself and thus to meet the nutritional requirements of pregnancy:

*“…because sometimes it’s not enough, so you have to think about other people as well that you are living with, so I’ll limit myself on certain things, because…ooh probably kids are coming from school, there is no money for bread, so let me leave the bread for them. See that type of thing. You limit yourself.” [29 year- old woman with 2 dependants, unemployed]*

While pregnant women in South Africa are exempt from paying fees for public-sector heath services, it is interesting that participants cited transport costs to get to facilities for antenatal care as an additional expense arising during pregnancy. Attendance at scheduled ANC visits was generally high but this was often achieved in spite of having no money to pay for transport – and therefore having to walk to the clinic, no matter the distance. Only one woman missed an appointment as a result of being too ill to attend, while two others missed their clinic appointments due to a lack of money. Another woman, interviewed at full term, who did not have the ZAR8, or US$1 needed for a taxi, described walking for 40 minutes to the clinic with her 11-month old baby on her back. Leaving the child with a neighbour would cost her around US$6 a day. Even with these constraints, she felt strongly that antenatal check-ups should not be missed:

*“…because I can’t lose a check- up, I have appointment with doctor, I have to go…” [27 year-old woman, with 1 dependant, HIV positive, unemployed]*

Other women echoed this sentiment, citing transportation as a major concern now that they were pregnant and in need of additional health care:

*“Transport money to come to the clinic costs a lot and also doctor’s charges, because sometimes I have to go to a specialist doctor as a result of my condition.” [26 year-old woman with two dependants, HIV positive, unemployed].*

*“I need a chance to go to the doctors, sometimes you find out that I’m sick, but I don’t have money to go to the doctors, or maybe I want to go to the clinic but I don’t have transport money, I just sit at home and take wrong tablets because I don’t have transport money” [38 year-old woman with three dependants, diabetic, unemployed]*

This concern extended especially to the need to get to the hospital at the onset of labour – a transport expense that could occur at any time of the day or night. Participants estimated the cost of hiring a private car in the middle of the night (when public transport was no longer available and ambulances were viewed as unreliable) for transportation to the hospital as between ZAR320-400 (US$40-50).

One factor further complicating women’s transport needs for antenatal visits relates to clinic times and quotas imposed by health workers. Nurses at RMH antenatal clinic start attending to patients at approximately 7 am and doctors’ consultations begin around 10 am; however, many patients arrive earlier than 7 am to obtain a number and take their place in the queue. One woman reported leaving home as early as 4:30 am, despite the fact that it only takes her 15 minutes to walk to the hospital. She explained that she needed to arrive early because:

*“… the nurses, the sisters come in and they start handing out the numbers and [if] you not here by seven o’clock you must go away.” [29 year old-woman with 2 dependants, unemployed]*

This quota system and the clinic’s restricted operating hours were mentioned by a number of participants as a significant inconvenience, and one that needed to be navigated carefully to avoid multiple visits, and therefore increased transport costs.

## Discussion

Though pregnancy is a normal life occurrence, it has the potential to further marginalise women and children living in already vulnerable households. Pregnancy poses considerable financial pressure on households, primarily through reducing maternal ability to work; increasing the volume and variety of food required to support pregnancy and breastfeeding; introducing travel costs for visits to health facilities, along with the costs of raising a new child. In this respect, our study concurs with evidence from a national survey in South Africa which provides compelling quantitative data on how the socio-economic vulnerability of poor women has substantial impacts on maternal health and wellbeing [21].

Most of the women interviewed for this study had limited means available for ensuring their own nutritional needs were met during pregnancy or for preparing for the arrival of a newborn. Costs associated with pregnancy coincided with the diminishing of their own ability to secure income and came at a time when partner support was mostly inconsistent, if provided at all. For many participants, this situation deepened their individual vulnerability and placed considerable pressure on already strained household resources. Where pregnancies are unplanned – as they were for the vast majority of women in our sample and, indeed, for women nationally [7] – households have to absorb a set of mostly unanticipated financial needs without the benefit of having budgeted for them in advance. The relatively late discovery of pregnancy (four months and later) among most of the women in our sample, a feature also found in other studies in South Africa [14, 15], is likely to further complicate financial planning at this challenging time.

Our findings on economic barriers to accessing antenatal care echo those reported in a recent study of the cost of maternal health services for women attending two urban and two rural clinics in South Africa [22, 23]. The average costs of childbirth for a household were ZAR320 (US$40), which was mainly for supplies and transport, while costs in rural areas were almost double that of urban areas. Not surprisingly, rural women had the greatest barriers to accessing delivery services, such as long travel times, higher costs, lower ability-to-pay and more sold household assets or borrowed money for these costs. On average, a third of total monthly household expenditure was spent on the direct costs of childbirth. Catastrophic health expenditure, defined as spending more than 10 % of monthly household expenditure on health, occurred in two-thirds of all women, with almost 90 % reporting this in the rural area of Bushbuckridge. Inability to meet the costs of private transport meant that many women relied on public-sector ambulances to collect them when in labour. As in our study, many recounted long waits for ambulances and even delivery while waiting for an ambulance to arrive. Elsewhere, it has been noted that the unpredictability of delivery outcomes and costs often makes budgeting for delivery difficult and may delay access to emergency care for women [24].

The need for more structural interventions to directly tackle economic vulnerability for this group cannot be ignored. Pregnant women in many LMICs presently receive little or no direct state support. While user fees have been removed for antenatal care and childbirth services, for many women, the financial costs involved in getting to the clinic for scheduled visits along with costs incurred by time off work needed to access these services can be substantial. Nationally, an estimated 17 % of women still deliver without a skilled birth attendant [17], and many women are either turned away from public sector facilities for attending “too early” or discouraged by long waiting times and patient quotas [14]. Global evidence suggests that ensuring access to skilled birth attendants and emergency obstetric care are two interventions critical to avoiding maternal deaths [25, 26], making these issues all the more urgent for the state to address.

An important policy question is thus whether the state should be providing specific maternity and early child support to poor women, in addition to the health system reform suggested above. This support, in the form of food parcels, transport vouchers, a cash grant, or a combination of such elements, has the potential to improve maternal nutritional status and pregnancy outcomes for mother and child, while at the same time developing synergistic linkages between health and social service departments [27, 28]. The Child Support Grant, introduced in 1998, has proven successful in reducing hunger, improving nutrition, and in promoting health and development in young children, among many other benefits [29, 30]. Based on the means test to determine eligibility for this grant, 471.3 % of pregnant women in South Africa would qualify. Yet the Child Support Grant is not intended for use by women to meet their own needs during pregnancy and in the postpartum. It also begins too late to help infants, especially during the most vulnerable periods of life: when the infant is still *in utero* and in the first few weeks and months after childbirth. Feminist economists and social theorists have provided powerful critiques of the state’s social welfare system and how it has failed women, who remain the primary – yet unacknowledged – caregivers of children [31–33]. From this perspective, as Hassim points out, the Child Support Grant effectively regards women as little more than the “conduits” for child-care assistance [34].

We would argue that it is not only the case that pregnant women’s needs should be met, but also that it is their legal and moral *right* to receive such social assistance, as pledged by the state in the South African Constitution and Bill of Rights. The state further has a constitutional obligation to implement positive measures to attain gender equality [6]. This includes improving the maintenance system, which continues to fail the more than half of mothers nationally who are single [7].

From a labour perspective, wage compensation has long been acknowledged as a right of the working woman to social support for her role in bearing and rearing children [35, 36]. In the formal employment sector, it is usual practice for women to be paid during a portion of pregnancy and a period thereafter (although in South Africa the benefit amount is equal to less than half of the woman’s salary, if paid by the state rather than private employers). That *all* women, whether employed or not, are not compensated for their time and labour in pregnancy and breastfeeding, is indicative of the continued invisibility of women’s overwhelming responsibility for raising and caring for children: labour critical for social reproduction. Our study highlights the extent to which this unpaid ‘caring work’ begins before the baby is even born, and pushes poor women further into situations of vulnerability in the absence of external support.

There are several important limitations to this study. Firstly, the client population at RMH is not representative of all pregnant women in the area, nor does the hospital exclusively serve women from poor households. In fact, poorer women more often access primary healthcare clinics and relatively wealthier ones attend tertiary hospitals, such as RMH [37]. The limited number of women interviewed for the study also restricts the extent to which generalisations may be made based on the findings. Also, a larger sample size may have allowed us to examine the study questions in greater detail. It is also possible that participants may have exaggerated descriptions of their financial situations to make them appear more extreme, particularly if they intuited that the interview was about potential provision of state support.

## Conclusion

Overall, levels of vulnerability and inequity are high in South Africa, as measured by health status such as HIV infection, or by socio-economic markers such as income, education level or access to housing. Each of these vulnerabilities is heightened among pregnant women. HIV is also a major health burden, despite relatively high levels of access to antiretroviral treatment, and has high financial costs [38, 39]. State support that implicitly or explicitly encourages early attendance at antenatal care would enhance interventions for prevention of mother to child transmission of HIV (PMTCT) through reducing delays in initiation of antiretroviral drugs [40]. The added health concerns associated with HIV and its impact on the need for good nutrition and higher calorie intake cannot be overlooked as many pregnant South Africans are faced with this condition [41].

In summary, the experience of poor pregnant women, as shown by the study findings reported here, rather than being a positive and natural part of the life cycle, is characterised by disempowerment, dependency and crisis. A rapidly emerging new set of priorities related to pregnancies, particularly in a context where most pregnancies are unplanned, has marked financial implications for households and for individual women. Malnutrition from a lack of diversity in diet in pregnancy, experienced by many participants in our study, enhances the risk of adverse pregnancy outcomes, and has potential long-term, inter-generational impacts. Extending the Child Support Grant to include the period of pregnancy would not only serve to acknowledge and address the particular challenges faced by poor women, but also go some way to securing the health of newborn children and future generations.

## Footnotes

1 The upper-bound poverty line refers to the food poverty line (the amount of money an individual will need in order to consume the required energy intake, i.e. ZAR305 or $36 per month in March 2009) plus the average amount derived from non-food items of households whose total food expenditure is equal to the food poverty line (Statistics South Africa 2012).

2 Women who contribute to the UIF are entitled to claim a maximum of six months maternity benefits, which amount to one week's maternity benefit for every six weeks worked (to claim the full six months of benefits, then, women have to have worked continuously in the formal sector for at least three years). Importantly, the benefit is equal to only 45 % of the woman's salary (Moolman 1997).

3 This amount was chosen as it approximates the current value of the Child Support Grant (ZAR300 a month per child; $36).

4 A single parent earning ZAR2900 (US$360) or less per month, or a married couple jointly earning ZAR5800 ($725) or less per month.
